# Systematic review of complications arising from male circumcision

**DOI:** 10.1002/bco2.123

**Published:** 2021-11-11

**Authors:** Stanca Iris Iacob, Richard S. Feinn, Lauren Sardi

**Affiliations:** ^1^ Frank H. Netter MD School of Medicine Quinnipiac University North Haven Connecticut USA; ^2^ Department of Sociology Quinnipiac University Hamden Connecticut USA

**Keywords:** circumcision, circumcision complications, male circumcision, neonatal circumcision, neonatal male circumcision

## Abstract

**Background:**

Neonatal male circumcision is the most common procedure performed on paediatric patients (Simpson et al., 2014) and one of the most common surgical procedures in the world (American Academy of Pediatrics, 2012).

**Methods:**

A search was conducted for articles about complications arising from male circumcision surgeries by entering the term ‘male circumcision’ into PubMed on June 16, 2020. Six thousand six hundred forty‐one articles published from 1945 to 2020 were found. Seventy‐eight articles were ultimately selected for the systematic review.

**Results:**

The 78 articles selected from the literature search were entered into one of three tables. The first table includes 15 articles pertaining to chart reviews and cohort studies and report complication rates. The second table reports specific complications from 51 case reports and case series, and the third table is a summary from 12 articles regarding physician questionnaires and society recommendations. Additionally, the 78 articles were used to compile a list of 47 specific complications arising from male circumcision surgeries.

**Conclusions:**

Complications from neonatal male circumcisions are common and healthcare providers need to be better informed of the potential complications of the surgery so that they can more effectively counsel their patients about potential risks, likelihood of complications and what can be done to prevent them. While experienced providers who practice in sterile settings have better outcomes with fewer complications, encouraging parents to take into account who is performing their son's circumcision, what was their training, how clean is their practice and how much experience they have and reminding them they have the option to decline the procedure entirely allow the parents to get a more complete picture and play an essential role in the decision‐making process.

## 1 INTRODUCTION

Neonatal male circumcision is the most common procedure performed on paediatric patients^1^ and one of the most common surgical procedures in the world.^2^ About 30% of all men are circumcised worldwide.^3^ In recent years, the rate of neonatal circumcision has been dropping in the United States, but it still varies among certain populations.^2^ Despite the prevalence of this procedure, there are a number of ethical and moral debates surrounding the practice of male circumcision,^4^ especially regarding whether a neonate can give consent for his own circumcision. In June 2012, an appellate court in Germany ruled that nontherapeutic circumcision of boys is irreversible bodily harm that violates the child's right to autonomy and self‐determination and that the procedure should be delayed until an age where the boy can consent for himself.^5^, ^6^ The discussion around male circumcision is often emotionally charged and intense, with some even comparing the practice to female genital mutilation.

Historically, male circumcision has been practiced since ancient times. There are reports of male circumcision on Egyptian mummies and in the biblical covenants recorded in the Old Testament.^1^ In the United States, male circumcision began to be widely practiced in the Victorian era as a means of deterring masturbation and improving genital cleanliness.^7^, ^8^

In modern times, male circumcision is performed at various ages by many potential providers, in many areas of the world, as part of cultural, religious and/or medical practices. Those of Jewish and Muslim faith consider male circumcision an indispensable element to their religion.^6^ Jewish people circumcise their boys on the eighth day of life.^9^ Muslim people circumcise their boys before puberty, generally between 4 and 13 years of age.^9^, ^10^

Parents commonly hear about the reported health benefits of male circumcision such as decreased rate of viral sexually transmitted infections, including HIV and HPV, among heterosexual males. However, spread of HIV and some other STIs is dependent on multiple risk factors, including unprotected sexual intercourse, intravenous drug use, unscreened blood donations and vertical transmission during gestation or childbirth. Massive circumcision efforts have begun in Africa to voluntarily circumcise males in order to try to limit the spread of HIV.^11^ The data regarding this are not well reported and does not provide resounding support to the idea that male circumcision prevents spread of HIV. Additionally, while male circumcision may be useful in protecting against the incidence of male urinary tract infections,^12^ bacterial colonization is still present after circumcision, so genital hygiene is regarded as more effective in preventing UTIs rather than circumcision surgeries.^13^ In certain instances where hygiene is poor, circumcision may be implemented to prevent urinary tract infections.

In the United States, parents are generally not informed how circumcisions are performed. Those that take place in the medical setting are done with the use of the Gomco, Mogen or Plastibell clamps. Additionally, ‘free‐hand circumcision’ is also practiced. Each instrument and technique has its own risks and benefits. Local anaesthesia is supposed to be administered for neonatal circumcisions, and general anaesthesia must be administered for circumcisions past the neonatal period.^14^ Circumcisions should ideally be done in sterile medical settings by trained and experienced providers due to the nature of this operation.

In some situations, male circumcision surgeries are necessary for medical reasons. Pathologic phimosis and recurrent balanitis are generally accepted medical indications for circumcision.^10^ Physiologic phimosis is present in almost all neonates, and the prepuce is eventually retractable in 99% of males by age 16.^15^, ^16^ If a prepubescent patient presents with tight but otherwise normal foreskin, watching and waiting until after puberty should be considered.^15^ A dorsal slit or circumcision surgery will eliminate the pathologic phimosis if it is bothersome to the patient, or the patient is postpubescent.

As with any surgical procedure, complications after a male circumcision surgery are possible. Some of these complications are minor and easily treated such as bleeding (in patients without a bleeding disorder) and infection; others, however, require additional surgery to correct the complication such as trapped penis and unsatisfactory cosmetic results. Some complications are irreversible such as decreased sexual sensation and death. Psychological issues have been reported to arise in children after operations, including circumcisions.^17^

The purpose of this research is to compile a recent and comprehensive list of complications that can arise from circumcision surgeries, in hopes that medical providers and parents will be able to make more informed decisions as to whether they will have their sons circumcised. The aim of the present study is to systematically review the studies on complications arising from male circumcisions published during the past two decades.

## 2 METHODS

A search was conducted for articles written regarding complications arising from male circumcision surgeries. The term ‘male circumcision’ was entered into PubMed on June 16, 2020, and 6641 articles published from 1945 to 2020 were found. The search was then narrowed to include only articles from 2000 to 2020, which contained 4464 papers. These papers were analysed to look for full‐text articles referring to specific complications arising from male circumcision surgeries, in English, Italian, Spanish, French, Portuguese or German languages. One hundred thirty‐four articles fit these criteria. These articles were read and ultimately 78 of them were used to compile a list of 47 specific complications arising from male circumcision surgeries. The article selection process is shown using the PRISMA flow diagram.

The 78 articles used to compile the list of 47 specific complications arising from male circumcision surgeries were entered into one of three tables. The first table organizes the articles that are chart reviews and cohort studies and gives estimates of the incidence of complications, the second table organizes the articles that are case reports and case series with the specific complications experienced, and the third table organizes the articles that are literature searches, physician questionnaires and society recommendations. Table 4 contains the list of 47 specific complications arising from male circumcision surgeries.

## 3 RESULTS

Seventy‐eight articles selected from the literature search were used to conduct the systematic review and build the above‐mentioned three tables as well as the comprehensive list of complications, contained in Table 4.

### 3.1 Chart reviews and cohort studies

Table 1 shows the 15 out of the 78 articles that were chart reviews and cohort studies. These followed a group of males that were circumcised and then saw how many of them developed complications from the circumcision operations. The studies were conducted mostly in the Middle East or United States, and the sample sizes ranged from as few as 25 patients^50^ to nearly 1.5 million infants.^69^ The complication rates ranged from less than 0.1% for meatal stenosis^69^ to almost 23% for bleeding in the context of a genetic condition: haemophilia, sickle cell trait and factor VII deficiency.^52^ The most common complication reported in most studies was haemorrhage/bleeding (outside of a genetic deficiency). Other complications reported included preputial stenosis, meatal stenosis, insufficient foreskin removal/redundant foreskin, long foreskin obstructing urine flow, early sloughing of foreskin tissue, device displacement, infection (minor and major), oedema, phimosis (referring to pathological phimosis), penile hematoma, bleeding in the context of a genetic condition (haemophilia, sickle cell trait and factor VII deficiency), skin bridges (penile skin adhesion), trapped/buried/concealed/inconspicuous penis, nonhealing wound, scrotal injuries and meatitis.

**TABLE 1:** Characteristics of chart reviews and cohort studies

Lead author	Year	Country	Study description	Patients age	Sample size	Type of complication	Number of complications	Complication rate
Tuncer	2017	Turkey	Male patients who underwent circumcisions between May 2014 and May 2015 in two separate paediatric surgery clinics were retrospectively analysed using the hospital registry system.	0–18 years	2062	Infection (minor and major)	2	1%
						Haemorrhage/bleeding (outside of a genetic deficiency)	11	
						Scrotal injuries	1	
						Meatitis	1	
						Trapped/buried/concealed/inconspicuous penis	6	
Hung	2018	USA	A longitudinal population analysis of the California Office of Statewide Health Planning and Development database between 2005 and 2010, calculating early and late complications of male circumcisions.	<5 years	24 432	Infection (minor and major)	50	1%
						Haemorrhage/bleeding (outside of a genetic deficiency)	171	
						Nonhealing wound	23	
Srinivasan	2015	USA	A retrospective chart review of patients circumcised at a well‐baby nursery, neonatal intensive care units (NICU), and special care nursery from 2007 to 2012.	Range of 0–144 days	7038	Haemorrhage/bleeding (outside of a genetic deficiency)	4	0.64%
						Meatal stenosis	3	
						Trapped/buried/concealed/inconspicuous penis	9	
						Insufficient foreskin removal/redundant foreskin	13	
						Skin bridges (penile skin adhesion)	16	
Elalfy	2012	Egypt	25 patients with known severe haemophilia A less than 36 months old were circumcised.	<3 years	25	Bleeding in the context of a genetic condition: haemophilia, sickle cell trait and factor VII deficiency	1	4%
Yilmaz	2010	Turkey	Retrospective review of medical records of 50 patients with haemophilia who underwent circumcision at their hospital according to Izmir protocol between 1996 and 2009.	13 months	50	Bleeding in the context of a genetic condition: haemophilia, sickle cell trait and factor VII deficiency	3	6%
Rodriguez	2009	USA	Search of the patient database for records of children who were followed up at the Mayo Clinic Comprehensive Hemophilia Center from 2000 through 2007 and who had been circumcised.	Birth to 7 years	48	Bleeding in the context of a genetic condition: haemophilia, sickle cell trait and factor VII deficiency	11	22.92%
Ferhatoglu	2019	Turkey	A review of 9 years of records from Bursa State Hospital	Mean = 94 months	1096	Haemorrhage/bleeding (outside of a genetic deficiency)	16	11.68%
						Penile hematoma	4	
						Oedema	108	
Kidger	2012	UK	A retrospective study was performed of outcomes of Plastibell circumcision in a community‐based circumcision service provided by trained paediatric surgeons.	n/a	560	Phimosis (secondary)	5	0.89%
Litwiller	2017	USA	Observational cohort study of 260 infants undergoing circumcision with Gomco clamp. Vitamin K was given to neonates at delivery. Demographic data, procedural characteristics, bleeding complications and interventions were recorded.	1–12 days	260	Haemorrhage/bleeding (outside of a genetic deficiency)	31	11.9%
Talini	2018	Brazil	Retrospective analysis of medical records of patients submitted to circumcision from May 1, 2015, to May 31, 2016, to evaluate post‐operative complications of circumcision requiring surgical reintervention.	5.27 years	2441	Infection (minor and major)	2	1.88%
						Haemorrhage/bleeding (outside of a genetic deficiency)	26	
						Preputial stenosis	18	
Heras	2018	USA	A retrospective chart review of all term neonates who had circumcision performed between August 2011 and December 2014 at two community hospitals in New York.	0–18 days	1064	Haemorrhage/bleeding (outside of a genetic deficiency)	41	3.85%
Plank	2013	Botswana	A case of probable vitamin K deficiency bleeding that occurred during a clinical trial of infant circumcision.	1–10 days	150	Haemorrhage/bleeding (outside of a genetic deficiency)	6	4%
Feinberg	2010	USA	A prospective cohort study of 537 consecutive Gomco circumcisions. The authors defined bleeding and operator experience, both current and long term, and sought to correlate them.	39.33 weeks	537	Haemorrhage/bleeding (outside of a genetic deficiency)	24	4.47%
Odoyo‐June	2015	Kenya	A PrePex implementation study in routine service delivery among 427 men in the age range of 18–49 in western Kenya.	18–49 years	427	Device displacement	5	2.8%
						Early sloughing of foreskin tissue	3	
						Long foreskin obstructing urine flow	2	
						Insufficient foreskin removal/redundant foreskin	2	
Morris	2017	Australia	A meta‐analysis of 27 studies (350 meatal stenosis cases among 1 498 536 males) found that the risk of meatal stenosis in circumcised males was 0.656%.	<1 year	1 498 536	Meatal stenosis	27	0.656%

### 3.2 Case reports and case series

Table 2 shows the 51 out of the 78 articles that were case reports and case series. These articles followed a group of males that had specific complications after their circumcision operations. Many articles discussed how the complications were managed. The studies were conducted in North America, Europe, Africa, Oceana, South America and Asia. The patient ages ranged from 1 day old^81^ to 55 years old.^31^ The most common complications reported were trapped/ buried/concealed/inconspicuous penis and haemorrhage/bleeding (outside of a genetic deficiency). Other complications reported included extensive penile skin defects/avulsion, necrosis, urethrocutaneous fistula, device displacement, infection (minor and major), partial penile/glans amputation, insufficient foreskin removal/redundant foreskin, glandular adhesion (of remnant foreskin), implantation dermoid/epidermal inclusion cysts/penile implantation cyst, chordee, (acute) ischaemia (which can lead to necrosis), pain, decreased urine output, penile herpes simplex virus type 1 infection (after Jewish ritual circumcision), necrosis, partial penile/glans amputation, complete penile amputation, iatrogenic hypospadias, skin bridges (penile skin adhesion), meatal stenosis, trapped/buried/concealed/inconspicuous penis, bleeding in the context of a genetic condition: haemophilia, sickle cell trait, factor VII deficiency, death (from unrecognized bleeding or infection), keloids, phimosis (referring to pathological phimosis), nonhealing wound, cicatrix, unsatisfactory cosmetic results including uncircumcised appearance, sudden infant death syndrome, scrotal injuries, sexual dysfunction, intraperitoneal bladder perforation (leading to life‐threatening renal failure), Fournier's gangrene, penile mycosis leading to penile gangrene, myiasis (fly infestation), denuded penis, lower clinical neurophysiological elicitability of the penilo‐cavernosus reflex and traumatic neuroma. Some complications are uniquely reported, such as denuded penis, lower clinical neurophysiological elicitability of the penilo‐cavernosus reflex, traumatic neuroma, myiasis (fly infestation), penile mycosis leading to penile gangrene, intraperitoneal bladder perforation (leading to life‐threatening renal failure), sexual dysfunction, sudden infant death syndrome, scrotal injuries, death (from unrecognized bleeding or infection), complete penile amputation, iatrogenic hypospadias, decreased urine output and chordee. Several articles referred to complications arising from male circumcisions from religious settings, such as penile herpes simplex virus type 1 infection (after Jewish ritual circumcision).

**TABLE 2:** Characteristics of case reports and case series

Lead author	Year	Country	Study description	Patient age	Types of complication
Gao	2019	China	Case report of a 31‐year‐old man with an extensive defect of the penile skin caused by a circumcision performed 20 days previously	31	Extensive penile skin defects/avulsion
Bode	2009	Nigeria	Prospective case series of penile injuries resulting from proximal migration of the Plastibell device in neonate boys. Twenty‐three injuries resulting from circumcision with the Plastibell device all occurred from prolonged retention of the ring.	10–27 (mean 14.7 ± 4.2)	Extensive penile skin defects/avulsion
					Necrosis
					Urethrocutaneous fistula
					Device displacement
Osifo	2009	Nigeria	A retrospective analysis of all cases of male children managed for circumcision mishaps between January 1998 and December 2007 at the University of Benin Teaching Hospital, Benin City, Nigeria. There were 346 male children aged between 6 days and 12 years with circumcision mishaps.	Between 6 days and 12 years	Extensive penile skin defects/avulsion
					Infection (minor and major)
					Partial penile/glans amputation
					Haemorrhage/bleeding (outside of a genetic deficiency)
					Urethrocutaneous fistula
					Device displacement
					Insufficient foreskin removal/redundant foreskin
					Glandular adhesion (of remnant foreskin)
					Implantation dermoid/epidermal inclusion cysts/penile implantation cyst
					Chordee
Migliorini	2016	Italy	Single patient underwent dorsal penile nerve block circumcision.	24 years	(Acute) Ischaemia (which can lead to necrosis)
					Pain
Gold	2015	Australia	Retrospective chart review of cases presenting with circumcision‐related problems to the Royal Children's Hospital, Melbourne, Australia, between 2012 and 2014. Over a 29‐month period, 167 children with a circumcision‐related emergency department presentation were identified.	Average 3 years	Infection (minor and major)
					Haemorrhage/bleeding (outside of a genetic deficiency)
					Pain
					Decreased urine output
Yossepowitch	2013	Israel	A case of direct orogenital suction performed during circumcision in the newborn period that resulted in herpes simplex virus type 1 infection which presented at 2.5 years of age.	2.5 years	Penile herpes simplex virus type 1 infection (after Jewish ritual circumcision)
Aminsharifi	2012	Iran	Two cases of severe glanular ischaemic necrosis that occurred after circumcision.	3.5 years	Necrosis
Pepe	2015	Italy	Case report of a young man with severe ischaemia of the glans penis following circumcision.	20 years	Necrosis
					(Acute) Ischaemia (which can lead to necrosis)
Appiah	2016	Ghana	Consecutive cases of circumcision‐related injuries seen at the unit over an 18‐month period at the Urology Unit of the Komfo Anokye Teaching Hospital in Kumasi. A total of 72 cases of circumcision‐related injuries were recorded during the 18‐month period.	2 day to 11 years old	Partial penile/glans amputation
					Complete penile amputation
					Urethrocutaneous fistula
					Iatrogenic hypospadias
					Insufficient foreskin removal/redundant foreskin
					Skin bridges (penile skin adhesion)
					Implantation dermoid/epidermal inclusion cysts/penile implantation cyst
Manentsa	2019	South Africa	Three cases of glans amputation in young healthy men that presented for voluntary medical male circumcision.	11.67 years	Partial penile/glans amputation
Ekenze	2013	Nigeria	A case series of 64 patients with complications of neonatal circumcision managed by surgery between June 2006 and May 2012 at the University of Nigeria Teaching hospital Enugu.	7.8 months	Partial penile/glans amputation
					Meatal stenosis
					Trapped/buried/concealed/inconspicuous penis
					Urethrocutaneous fistula
					Glandular adhesion (of remnant foreskin)
					Implantation dermoid/epidermal inclusion cysts/penile implantation cyst
Charlesworth	2011	UK	The case of a 4‐year‐old boy who, shortly after a Plastibell circumcision, with the ring still in situ, experienced trauma to his glans, resulting in complete amputation.	4 years	Partial penile/glans amputation
Bocquet	2010	France	The cases of six children, seen over 1 year at the emergency department for bleeding complication or mutilation after ritual home circumcision.	From 7 days to 4 years	Partial penile/glans amputation
					Haemorrhage/bleeding (outside of a genetic deficiency)
Ademuyiwa	2012	Nigeria	Retrospective study of all cases with complications of circumcision who were managed in the Pediatric Surgery Unit of the Lagos University Teaching Hospital between 2008 and 2010. Thirty‐six patients had circumcision complications.	2 days and 9 years (median‐3 months)	Partial penile/glans amputation
					Haemorrhage/bleeding (outside of a genetic deficiency)
					Meatal stenosis
					Trapped/buried/concealed/inconspicuous penis
					Urethrocutaneous fistula
					Skin bridges (penile skin adhesion)
					Implantation dermoid/epidermal inclusion cysts/penile implantation cyst
Banihani	2014	USA	A case of complete penile amputation at the penopubic junction using a Mogen clamp in a 7‐day‐old neonate with replantation using post‐operative leech therapy.	7 days	Complete penile amputation
Maataoui	2018	Morocco	The case of two brothers with sickle cell trait and major haemophilia A.	1.75 years	Bleeding in the context of a genetic condition: haemophilia, sickle cell trait and factor VII deficiency
Mense	2018	Canada	A case of a healthy male neonate born at term, circumcised on Day 1 of life. Facing ongoing bleeding at the incision site, further investigations revealed a diagnosis compatible with severe haemophilia A. He deteriorated on Day 2.		Bleeding in the context of a genetic condition: haemophilia, sickle cell trait and factor VII deficiency
					Death (from unrecognized bleeding or infection)
Ettarfaoui	2017	Morocco	The case of a newborn of parents who had first‐degree consanguinity, admitted to hospital on Day 10 of life with postcircumcision haemorrhagic syndrome.	10 days	Bleeding in the context of a genetic condition: haemophilia, sickle cell trait and factor VII deficiency
Mansouritorghabeh	2013	Iran	Retrospective study of cases from information using evaluation medical records in three major hospitals during last 15 years and list of patients with bleeding disorders obtained from a haemophilia centre, including data on doing circumcision or not, types of treatment before and post the procedure and occurrence of bleeding episodes after the surgery. 424 cases with various common and rare bleeding disorders who had circumcised were found.	25.07 ± 1.44 years	Bleeding in the context of a genetic condition: haemophilia, sickle cell trait and factor VII deficiency
Galukande	2015	Uganda	Three cases were described of previously undiagnosed haemophilia A males circumcised during routine voluntary medical male circumcision service delivery.	20.67	Bleeding in the context of a genetic condition: haemophilia, sickle cell trait and factor VII deficiency
Alyami	2019	Canada	A retrospective review of six keloid cases that had developed after genital procedures, including male circumcision, between 2000 and 2017 was conducted.	13 years	Keloids
Cappuyns	2019	Malawi	A case of a 13‐year‐old boy with penile keloids following traditional circumcision.	13 years	Keloids
Demirdover	2013	Turkey	A case of keloid formation after circumcision.	3 years	Keloids
Özdemir	2019	Turkey	The medical records of 25 boys with postcircumcision secondary phimosis were reviewed.	2–5 years	Phimosis (secondary)
Fekete	2010	Hungary	Forty‐eight men who underwent circumcision between August 2005 and December 2008 were referred to the practice with dissatisfied results for treatment.	27.4 years	Phimosis (secondary)
					Nonhealing wound
					Cicatrix
					Unsatisfactory cosmetic results including uncircumcised appearance
					Insufficient foreskin removal/redundant foreskin
Elhaik	2018	UK	Collation of latitudinal data from 15 countries and 40 US states sampled during 2009 and 2013, using linear regression analyses and likelihood ratio tests to calculate the association between SIDS and male neonatal circumcision (MNC) and prematurity.	0–12 months	Sudden infant death syndrome
Bar‐Yosef	2019	Israel	Reports of all circumcision complications between 2007 and 2014 were evaluated for Scrotal injuries during neonatal circumcision.	n/a	Scrotal injuries
Özen	2018	Turkey	Physical examination, lower urinary tract symptoms, urethral meatus configuration and surgical procedures of 18 children admitted for routine circumcision, who had congenital hooded prepuce with normally located urethral meatus, were analysed.	6 years	Meatal stenosis
Özen	2017	Turkey	Records of children who had meatoplasty between 2014 with 2016 were analysed retrospectively. Only children with MS who had newborn circumcision performed in our clinic were included in the study.	52.5 ± 17.9 months	Meatal stenosis
Joudi	2010	Iran	Male children who had been circumcised during the neonatal period and presented at the author's paediatric clinic for reasons other than urinary complaints were examined and interviewed regarding urination problems.	5–10 years	Meatal stenosis
Homer	2014	UK	Medical records of boys with clinical lichen sclerosis were reviewed for the period 2000 to 2010. After circumcision for lichen sclerosis up to one in five boys requires a subsequent operation for meatal pathology.	9 years	Meatal stenosis
Alpert	2018	USA	A retrospective review of the records of all patients who presented to the outpatient clinic since 2014 with cicatrix formation after neonatal circumcision.	2.7 months	Cicatrix
Tempark	2013	USA	Eleven cases of boys who presented with dermatologic complications of circumcision in an outpatient paediatric dermatology clinic.	4.28 years	Phimosis (secondary)
					Trapped/buried/concealed/inconspicuous penis
					Skin bridges (penile skin adhesion)
					Glandular adhesion (of remnant foreskin)
					Implantation dermoid/epidermal inclusion cysts/penile implantation cyst
Ignjatović	2010	Serbia	Case report of excessive circumcision with complete resection of the penile skin is shown.	55 years	Infection (minor and major)
					Trapped/buried/concealed/inconspicuous penis
					Sexual dysfunction
Isik	2010	Turkey	A case of congenital buried penis with deteriorated clinical findings after two circumcision procedures.	1.5 years	Trapped/buried/concealed/inconspicuous penis
					Insufficient foreskin removal/redundant foreskin
Storm	2011	USA	Evaluation of 51 patients with penile adhesions and hidden penis after newborn circumcision was compared with 33 age‐matched controls. Boys with hidden penis had a statistically higher weight for length percentile at birth and at urological evaluation.	16.5 months	Trapped/buried/concealed/inconspicuous penis
					Skin bridges (penile skin adhesion)
Eroglu	2009	Turkey	During a routine visit to the paediatrician 88 infants with buried penis were assessed by a single paediatric surgeon between January 2004 and June 2007. In December 2007, all of these children were re‐examined by the same paediatric surgeon, and the natural growth of the genitalia was analysed.	3.3 months	Trapped/buried/concealed/inconspicuous penis
Sancaktutar	2011	Turkey	An 18‐year‐old boy presented with urine passage from four fistula orifices. He had been circumcised by nonmedical personnel when he was 2 years old. During the surgery, after degloving the penis, it was observed that the fistulae tracts were combining. There were only two fistulae orifices on the urethra. The fistulae were repaired with simple closure. This is the second case reported in the literature describing multiple urethrocutaneous fistulae.	18 years	Urethrocutaneous fistula
Dwyer	2016	USA	The case of an infant who suffered intraperitoneal bladder perforation secondary to routine neonatal circumcision with the Plastibell device.	1 day	Intraperitoneal bladder perforation (leading to life‐threatening renal failure)
Mittino	2018	Italia	The case of a 33‐year‐old patient who underwent circumcision at our institution and, 24 h after the procedure, developed an acute ischaemia of the glans.	33 years	(Acute) Ischaemia (which can lead to necrosis)
Cárdenas Elías	2016	Spain	A 10‐year‐old patient who underwent circumcision and a dorsal penile nerve block DPNB presents signs of penile ischaemia 2 h after surgery without any other symptoms.	10 years	(Acute) Ischaemia (which can lead to necrosis)
Sliwinski	2014	Australia	A 24‐year‐old man was admitted to ICU 10 days after elective circumcision with Fournier's gangrene.	24 years	Fourier's gangrene
Galukande	2014	Uganda	A 19‐year‐old male who had voluntary medical male circumcision performed using the dorsal slit technique and a 52‐year‐old male who had voluntary medical male circumcision performed with the sleeve resection method both developed Fourier's gangrene.	35.5 years	Fourier's gangrene
Hombalkar	2013	India	A 12‐year‐old boy with penile mycosis leading to penile gangrene.	12 years	Penile mycosis leading to penile gangrene
El‐Shazly	2012	Kuwait	The case of a 32‐year‐old patient with a hard lesion on the ventral aspect of penile skin. Examination revealed subcoronal cyst with hard stones and two small urethrocutaneous fistulae.	32 years	Urethrocutaneous fistula
					Implantation dermoid/epidermal inclusion cysts/penile implantation cyst
Saini	2010	India	A case of epidermal inclusion cyst of penis in a 5‐year‐old boy, as a late complication of circumcision.	5 years	Implantation dermoid/epidermal inclusion cysts/penile implantation cyst
Okeke	2009	Nigeria	A 10‐year‐old boy was seen at the urology outpatient clinic presenting with a globular swelling in the penile skin following circumcision.	10 years	Implantation dermoid/epidermal inclusion cysts/penile implantation cyst
Hossain	2012	Bangladesh	A 10‐year‐old boy presented with severe pain in his penile region following circumcision 7 days after. After examination, unhealthy granulation tissue was seen and maggots started coming out from the site of infestation, indicating presence of more maggots underneath the skin. An emergency operation was carried out to remove the maggots, and reconstruction was carried out at the plastic surgery department.	10 years	Myiasis (fly infestation)
Sinha	2012	UK	A 3‐month‐old boy, who underwent a ritual circumcision in the neonatal period	3 months	Necrosis
					Denuded penis
Podnar	2011	Slovenia	Confirmation of lower clinical neurophysiological elicitability of the penilo‐cavernosus reflex in circumcised men and in men with foreskin retraction. Out of a total of 247 men in the study, 31 were circumcised and 15 had retraction of the foreskin.	48 years	Lower clinical neurophysiological elicitability of the penilo‐cavernosus reflex
Cardoso	2015	Brazil	A 22‐year‐old patient, who had been circumcised at 8 years, presented with traumatic neuroma.	22 years	Traumatic neuroma

In spite of such large diversity of complications following circumcision, several observations can be made. The number of complications tends to be higher if circumcision is performed later in life (outside of the neonatal period), if it does not respect strict antisepsis (with geographic specifics), if it is performed by less experienced professionals, if it is conducted in religious setting (by non‐professionals) and if no clinical investigation is performed for identification of genetic bleeding defects.

### 3.3 Literature searches, physicians questionnaires and society recommendations

Table 3 shows the 12 out of the 78 articles that were literature searches, the results of physician questionnaires or society recommendations. These either analysed published articles on this subject, questioned physicians in how they perform circumcisions and treat various complications or were the declaration of various medical societies on their stances on male circumcisions. The articles were written in Europe, United States, Canada and the Middle East. Types of complications discussed included infection (minor and major), death (from unrecognized bleeding), unsatisfactory cosmetic results including uncircumcised appearance, partial penile/glans amputation, haemorrhage/bleeding (outside of a genetic deficiency), meatal stenosis, necrosis, partial penile/glans amputation, phimosis (referring to pathological phimosis), meatitis, trapped/buried/concealed/inconspicuous penis, urethrocutaneous fistula, iatrogenic hypospadias, insufficient foreskin removal/redundant foreskin, pain, skin bridges (penile skin adhesion), implantation dermoid/epidermal inclusion cysts/penile implantation cyst, chordee, suture sinus tracts, penile herpes simplex virus type 1 infection (after Jewish ritual circumcision) and bleeding in the context of a genetic condition: haemophilia, sickle cell trait, factor VII deficiency, oedema, nonhealing wound, circulatory shock, pain, excessive skin removal and injury to urethra.

**TABLE 3:** Characteristics of literature searches, physicians questionnaires and society recommendations

Lead author	Year	Country	Study description	Types of complication	Major findings/conclusions
Sorokan	2015	Canada	Canadian Pediatric Society position statement on newborn male circumcision.	Infection (minor and major)	While there may be a benefit for some boys in high‐risk populations and circumstances where the procedure could be considered for disease reduction or treatment, the Canadian Pediatric Society does not recommend the routine circumcision of every newborn male.
				Death (from unrecognized bleeding)	
				Unsatisfactory cosmetic results including uncircumcised appearance	
Brook	2016	USA	A literature search was conducted through June 25, 2015. The following search terms were used: circumcision, infections (all types), complication, wound infection, bacteremia, tetanus and guidelines.	Infection (minor and major)	Infectious complications following circumcision should be reduced with trained and competent practitioners performing the procedure using sterile techniques.
Morris	2016	Canada	A risk–benefit analysis of the Canadian Academy of Pediatrics Position Statement on newborn male circumcision.	Infection (minor and major)	The 2015 Canadian Pediatric Society position statement on *early infant (newborn) male circumcision* is at odds with the evidence. The Canadian Pediatric Society conclusions stem from errors in its risk–benefit analysis. In light of our findings, we recommend the Canadian Pediatric Society issue a revised statement.
				Partial penile/glans amputation	
				Haemorrhage/bleeding (outside of a genetic deficiency)	
				Meatal stenosis	
				Death (from unrecognized bleeding or infection)	
Krill	2011	USA	Description of how/when circumcisions are performed and various complications that can arise.	Infection (minor and major)	A thorough and complete preoperative evaluation, focusing on bleeding history and birth history, is imperative. Proper selection of patients based on age and anatomic considerations as well as proper sterile surgical technique are critical to prevent future circumcision‐related adverse events.
				Necrosis	
				Partial penile/glans amputation	
				Phimosis (secondary)	
				Haemorrhage/bleeding (outside of a genetic deficiency)	
				Meatal stenosis	
				Meatitis	
				Trapped/buried/concealed/inconspicuous penis	
				Urethrocutaneous fistula	
				Iatrogenic hypospadias	
				Insufficient foreskin removal/redundant foreskin	
				Pain	
				Skin bridges (penile skin adhesion)	
				Implantation dermoid/epidermal inclusion cysts/penile implantation cyst	
				Chordee	
				Suture sinus tracts	
Schofer	2015	Germany	Electronic databases were searched for articles about the infection risks of foreskin surgery, and the efficacy of circumcision in reducing the risks of sexual transmission of HIV, herpes viruses, HPV, *Treponema pallidum*, chlamydia, *Hemophilus ducrey* and *Neisseria gonorrhoeae*.	Infection (minor and major)	Neonatal circumcisions (and circumcision in early childhood) are irreparable interventions in the physical integrity, with very few medical indications. The risk of complications is dependent on the education of the circumciser (ritual and medical), analgesia and hygiene. Circumcisions should be performed under optimal surgical and hygienic conditions in informed and self‐determined young men only. In adolescents and adults, circumcision reduces the risk of the transmission of viral STIs (HIV, HSV and HPV), and there is also probably some effect on the sexual transmission of *T. pallidum and Haemophilus ducreyi* (insufficient, controversial data). The role of circumcision as an effective procedure to reduce the transmission of STIs is still under discussion, because important additional factors like sexual risk behaviour (e.g., unprotected sexual intercourse and promiscuity) have a strong influence on STI epidemiology.
				Haemorrhage/bleeding (outside of a genetic deficiency)	
				Meatitis	
Koren	2013	Israel	A review of the medical records of neonatal herpes simplex virus infection looking for cases who were born between January 2001 and December 2007 in five medical centres located in central Israel to determine the incidence and the clinical characteristics of neonatal herpes simplex virus infection in Israel.	Penile herpes simplex virus type 1 infection (after Jewish ritual circumcision)	The incidence of neonatal herpes simplex virus infection in Israel was found to be similar to the lower part of the scale reported in the United States, however, higher than the incidence reported in Canada or in Europe. Similar to more recent reports, our series demonstrates the shift toward the predominance of HSV‐1 in neonatal herpes simplex virus infection. In addition, none of the mothers in our series had a previous history of genital herpes. This study emphasizes the need for awareness of HSV infection in Israeli neonates.
Svoboda	2017	USA	Article discussing the ethics of Nontherapeutic Circumcision of Minors.	Necrosis	A comparison of benefits and risks is not ethically sufficient in an analysis of a nontherapeutic procedure performed on patients unable to provide informed consent; and that circumcision violates clinicians' imperatives to respect patients' autonomy, to do good, to do no harm and to be just. When due consideration is given to these values, the balance of factors suggests that NTC should be deferred until the affected person can perform his own cost–benefit analysis, applying his mature, informed preferences and values.
				Partial penile/glans amputation	
				Phimosis (secondary)	
				Meatal stenosis	
				Meatitis	
				Trapped/buried/concealed/inconspicuous penis	
				Urethrocutaneous fistula	
				Iatrogenic hypospadias	
				Implantation dermoid/epidermal inclusion cysts/penile implantation cyst	
Kearney	2015	USA	A survey of paediatric haematologists from Hemophilia Treatment Centers (HTC) across the United States to better understand the attitudes toward and management of neonatal circumcision in haemophilia patients.	Bleeding in the context of a genetic condition: haemophilia, sickle cell trait and factor VII deficiency	The recent shift in policy statements by professional societies in the United States regarding neonatal circumcision may result in more parents requesting this procedure during newborn period for its medical benefits. Paediatric haematologists must be prepared to address this while considering the risks inherent to persons with an underlying bleeding disorder. The study provides a foundation for future research regarding the optimal management and outcomes of neonatal circumcision in haemophilia to develop evidence‐based guidelines for the management of circumcision in this unique population.
Simpson	2014	USA	Review summarizing historical, cultural and ethical factors in neonatal circumcision and briefly compare common surgical techniques including anaesthesia.	Haemorrhage/bleeding (outside of a genetic deficiency)	We agree with the AAP policy that neonatal circumcision has medical benefits that exceed the medical risks and should be available for families who choose the procedure. We strongly support additional anticipatory guidance and documentation of informed consent. Cultural, religious and ethical family traditions must be respected and supported as physicians counsel these new families. We believe that neonatal circumcision is cost effective. Insurers including state associated Medicaid programmes should cover this procedure with adequate funds to encourage practitioners to perform neonatal circumcisions in appropriate settings. Additionally, we believe that a standardized circumcision curriculum is helpful for all resident training programmes whose graduates may expect to either perform neonatal circumcision or interact with parents during the prenatal and neonatal periods. Finally, physicians performing the procedure have a responsibility to demonstrate ongoing competency, including adequate pain control, during the procedure.
				Meatal stenosis	
				Unsatisfactory cosmetic results including uncircumcised appearance	
				Skin bridges (penile skin adhesion)	
Srinivasan	2011	USA	Provide a definition and description of inconspicuous penis and then to describe its management including surgical correction.	Trapped/buried/concealed/inconspicuous penis	Inconspicuous penis is a very common condition presenting to a paediatric urologist for surgical correction. Reconstruction is warranted in appropriate cases to avoid future psychosexual issues and provide the child with normal functional anatomy. Although the classification system to an extent is artificial and considerable overlap is present, it is useful in determining the primary anatomic issue and thus determines treatment. Diagnosis should be made on anatomical considerations and treatment individualized to the patients based on residual anatomy, type of deformity and the amount of skin cover available.
Cimador	2015	Italy	Management of inconspicuous penis in children.	Trapped/buried/concealed/inconspicuous penis	Inconspicuous penis is more common than is usually appreciated and often requires evaluation by a paediatric urologist. This disorder can have iatrogenic causes resulting from adhesions that are secondary to circumcision. If the inconspicuous penis is caused by abnormalities of the surrounding structures, it is essential to identify the primary anatomical issue. Diagnosis should be made on the basis of penile appearance while considering the anatomy of the penis. Treatment should be individualized to each patient on the basis of residual anatomy, type of deformity and the amount of covering skin available.
Edler	2016	Sweden	Data on significant complications following circumcision on boys under the age of one in Scandinavia over the last 20 years were collected. A systematic review was performed of fatal cases in the literature. Thirty‐two boys had cases that had been reported to the health authorities in the three countries, and a total of 74 complications were identified in these cases.	Infection (minor and major)	Complications following male circumcision in Scandinavia were relatively rare, but serious complications did occur. Based on the analyses of the severe cases, we argue that circumcision should only be performed at hospitals with 24‐h emergency departments.
				Necrosis	
				Oedema	
				Haemorrhage/bleeding (outside of a genetic deficiency)	
				Nonhealing wound	
				Trapped/buried/concealed/inconspicuous penis	
				Circulatory shock	
				Death (from bleeding or infection)	
				Insufficient foreskin removal/redundant foreskin	
				Pain	
				Excessive skin removal	
				Injury to urethra	

Positions taken on male circumcision by various groups could not be further apart. Most agree, though, that neonatal circumcision is an irreversible intervention in the physical integrity of the male.^94^ Humanists concentrate on the moral aspects related to the procedure, while physicians on the scientific ones. A categorical position is adopted by Andrew L. Freedman, a former member of the American Academy of Pediatrics Task Force on Circumcision, as he conceded that circumcision is fundamentally a religious or cultural practice in search of a ‘medical’ justification.^4^ At opposing end of the debate stands the Center of Disease Control (CDC) and the American Academy of Pediatrics (AAP), both concluding that the benefits of neonatal circumcision greatly outweigh the risks, suggesting at the same time, that the procedure be made available to informed parents asking for it for their sons.^95^ Situating itself somewhere equidistantly between these two poles, the Canadian Pediatric Society considers that the medical risk: Benefit ratio of routine newborn male circumcision is closely balanced and that it is challenging to make definitive recommendations for the entire male newborn population in Canada.^82^ A similar tone is expressed for the Scandinavian experience on circumcision.^32^

As an accepted frequent surgical procedure, several ancillary aspects are amply discussed, like, for instance, anaesthesia, related issues for circumcision in boys of various ages.^96^ Another aspect refers to preoperative and post‐operative therapy for children with known haemophilia (mainly factor VIII and IX deficiencies) undergoing circumcision. Fibrinolytic agents or coagulation factor replacements are among the most used and insure safe surgical procedures in specialized health facilities.^53^ Lastly, specific aspects of infections arising in connection to circumcision are discussed, emphasizing recommendations for strict asepsis during such operations^22^ and avoidance of ritual‐related HSV infections.^34^

Fewer complications occur if the circumcisions are performed by trained professionals in hospital settings versus other providers in ritual circumcisions. Recommendations and ethical issues raised include who performs circumcisions, what type of training they have, in which setting is the surgery performed, in what type of cleanliness conditions, with the presence of anaesthesia, how diagnoses of various complications should be made and why it is important to make them, the importance of cultural, religious and ethical family traditions in a family's decision to circumcise their son or not, the importance of being prepared to diagnose a bleeding disorder in a child during his circumcision operation and respecting a patient's autonomy in terms of his own circumcision.

### 3.4 Possible complications arising from male circumcision surgeries

Table 4 shows 47 unique complications arising from male circumcisions that were found among the 78 articles. The most common complications found in the articles were infection (minor and major), partial penile/glans amputation, bleeding in the context of a genetic condition: haemophilia, sickle cell trait, factor VII deficiency, haemorrhage/bleeding (outside of a genetic deficiency), preputial stenosis, meatal stenosis, trapped/buried/concealed/inconspicuous penis, urethrocutaneous fistula, insufficient foreskin removal/redundant foreskin and implantation dermoid/epidermal inclusion cysts/penile implantation cyst. The complications mentioned less often among the articles were penile hematoma, sudden infant death syndrome, intraperitoneal bladder perforation (leading to life‐threatening renal failure), circulatory shock, early sloughing of foreskin tissue, long foreskin obstructing urine flow, penile mycosis leading to penile gangrene, myiasis (fly infestation), denuded penis, suture sinus tracts, lower clinical neurophysiological elicitability of the penilo‐cavernosus reflex, sexual dysfunction, traumatic neuroma and decreased urine output.

**TABLE 4:** Possible complications arising from male circumcision surgeries

Complication	Description
1. Extensive penile skin defects/avulsion	Penile resurfacing using a reverse bilateral anterior scrotal artery flap^18^ Penile injuries from proximal migration of the Plastibell circumcision ring^19^ Circumcision mishaps in Nigerian children^20^
2. Infection (minor and major)	Newborn male circumcision^21^ Infectious complications of circumcision and their prevention^22^ Examination of short and long term complications of thermocautery, plastic clamping, and surgical circumcision techniques^23^ A longitudinal population analysis of cumulative risks of circumcision^24^ Acute ischemia of the glans penis after circumcision treated with hyperbaric therapy and pentoxifylline: Case report and revision of the literature^25^ Canadian Pediatrics Society position statement on newborn circumcision: A risk‐benefit analysis revisited^26^ Complications of circumcision^27^ Complications following circumcision: Presentations to the emergency department^28^ Circumcision mishaps in Nigerian children^20^ Rates of complications after newborn circumcision in a well‐baby nursery, special care nursery, and neonatal intensive care unit^29^ Male circumcision from an infectiological point of view^6^ Circumcision: Postoperative complications that required reoperation^30^ Reconstruction of the penile skin loss due to ‘radical’ circumcision with a full thickness skin graft^31^ Serious complications in male infant circumcisions in Scandinavia indicate that this always be performed as a hospital‐based procedure^32^
3. Penile herpes simplex virus type 1 infection (after Jewish ritual circumcision)	Penile herpes simplex virus type 1 infection presenting two and a half years after Jewish ritual circumcision of an infant^33^ Neonatal herpes simplex virus infections in Israel^34^
4. Necrosis	Nontherapeutic circumcision of minors as an ethically problematic form of iatrogenic injury^4^ Delayed glans necrosis after circumcision: Role of testosterone in salvaging glans^35^ Complications of circumcision^27^ Penile injuries from proximal migration of the Plastibell circumcision ring^19^ Ischemia of the glans penis following circumcision: Case report and revision of the literature^36^ Serious complications in male infant circumcisions in Scandinavia indicate that this always be performed as a hospital‐based procedure^32^ Penile resurfacing for denuded penis following circumcision^37^
5. Partial penile/glans amputation	Circumcision‐related tragedies seen in children at the Komfo Anokye Teaching Hospital, Kumasi, Ghana^38^ Nontherapeutic circumcision of minors as an ethically problematic form of iatrogenic injury^4^ Complications of high volume circumcision: Glans amputation in adolescents; a case report^39^ Canadian Pediatrics Society position statement on newborn circumcision: A risk‐benefit analysis revisited^26^ Complications of neonatal circumcision requiring surgical intervention in a developing country^40^ Complications of circumcision^27^ Surgical repair of traumatic amputation of the glans^41^ Bleeding complications after ritual circumcision: About six children^42^ Surgically correctable morbidity from male circumcision: Indications for specialist surgical care in Lagos^43^ Circumcision mishaps in Nigerian children^20^
6. Complete penile amputation	Complete penile amputation during ritual neonatal circumcision and successful replantation using postoperative leech therapy^44^ Circumcision‐related tragedies seen in children at the Komfo Anokye Teaching Hospital, Kumasi, Ghana^38^
7. Penile haematoma	Evaluation of male circumcision: Retrospective analysis of one hundred and ninety‐eight patients^45^
8. Bleeding in the context of a genetic condition: haemophilia, sickle cell trait and factor VII deficiency	Sickle cell trait and haemophilia: A rare association^46^ A newborn with simmering bleeding after circumcision^47^ Déficit congénital en facteur VII révélé par une hémorragie post circoncision^48^ Circumcision in males with bleeding disorders^49^ Risk of bleeding and inhibitor development after circumcision of previously untreated or minimally treated severe hemophilia A children^50^ A single centre experience in circumcision of haemophilia patients: Izmir protocol^51^ To circumcise or not to circumcise? Circumcision in patients with bleeding disorders^52^ Neonatal circumcision in severe haemophilia: A survey of paediatric haematologists at United States Hemophilia Treatment Centers^53^ A rare but important adverse event associated with adult voluntary medical male circumcision: Prolonged bleeding^54^
9. Oedema	Evaluation of male circumcision: Retrospective analysis of one hundred and ninety‐eight patients^45^ Serious complications in male infant circumcisions in Scandinavia indicate that this always be performed as a hospital‐based procedure^32^
10. Keloids	Keloid formation after pediatric male genital surgeries: An uncommon and difficult problem to manage^55^ A rare presentation of penile keloids after traditional circumcision: Case report^56^ Keloid formation after circumcision and its treatment^57^
11. Phimosis (referring to pathological phimosis)	Nontherapeutic circumcision of minors as an ethically problematic form of iatrogenic injury^4^ Secondary phimosis after circumcision^58^ Acquired phimosis after plastibell circumcision: A preventable consequence^59^ Complications of circumcision^27^ Revisions after unsatisfactory adult circumcisions^60^ Dermatological complications of circumcision: Lesson learned from cases in a pediatric dermatology practice^61^
12. Sudden infant death syndrome	Neonatal circumcision and prematurity are associated with sudden infant death syndrome (SIDS)^62^
13. Haemorrhage/bleeding (outside of a genetic deficiency)	Circumcision bleeding complications: Neonatal intensive care infants compared to those in the normal newborn nursery^63^ Circumcision: Postoperative complications that required reoperation^30^ Immediate complications of elective newborn circumcision^64^ A longitudinal population analysis of cumulative risks of circumcision^24^ Evaluation of male circumcision: Retrospective analysis of one hundred and ninety‐eight patients^45^ Acute ischemia of the glans penis after circumcision treated with hyperbaric therapy and pentoxifylline: Case report and revision of the literature^25^ Canadian Pediatrics Society position statement on newborn circumcision: A risk‐benefit analysis revisited^26^ Neonatal circumcision: New recommendations & implications for practice^1^ Vitamin K deficiency bleeding and early infant male circumcision in Africa^65^ Complications of circumcision^27^ Bleeding complications after ritual circumcision: About six children^42^ Complications following circumcision: Presentations to the emergency department^28^ Surgically correctable morbidity from male circumcision: Indications for specialist surgical care in Lagos^43^ Circumcision mishaps in Nigerian children^20^ Rates of complications after newborn circumcision in a well‐baby nursery, special care nursery, and neonatal intensive care unit^29^ Bleeding at circumcision: Patient or operator issue^66^ Male circumcision from an infectiological point of view^6^ Examination of short and long term complications of thermocautery, plastic clamping, and surgical circumcision techniques^23^ Serious complications in male infant circumcisions in Scandinavia indicate that this always be performed as a hospital‐based procedure^32^
14. Nonhealing wound	A longitudinal population analysis of cumulative risks of circumcision^24^ Revisions after unsatisfactory adult circumcisions^60^ Serious complications in male infant circumcisions in Scandinavia indicate that this always be performed as a hospital‐based procedure^32^
15. Scrotal injuries	Examination of short and long term complications of thermocautery, plastic clamping, and surgical circumcision techniques^23^ Scrotal injuries during neonatal circumcision^67^
16. Preputial stenosis	Circumcision: Postoperative complications that required reoperation^30^
17. Meatal stenosis	Complication of newborn circumcision: Meatal stenosis or meatal web^68^ Does circumcision increase meatal stenosis risk?—A systematic review and meta‐analysis^69^ Nontherapeutic circumcision of minors as an ethically problematic form of iatrogenic injury^4^ Circumcision: Postoperative complications that required reoperation^30^ Are mechanical and chemical trauma the reason of meatal stenosis after newborn circumcision^70^ Canadian Pediatrics Society position statement on newborn circumcision: A risk‐benefit analysis revisited^26^ Neonatal circumcision: New recommendations & implications for practice^1^ Complications of neonatal circumcision requiring surgical intervention in a developing country^40^ Complications of circumcision^27^ Incidence of asymptomatic meatal stenosis in children following neonatal circumcision^71^ Surgically correctable morbidity from male circumcision: Indications for specialist surgical care in Lagos^43^ Rates of complications after newborn circumcision in a well‐baby nursery, special care nursery, and neonatal intensive care unit^29^ Meatal stenosis in boys following circumcision for lichen sclerosus (balanitis xerotica obliterans)^72^
18. Cicatrix	Combination treatment for cicatrix after neonatal circumcision: An office‐based solution to a challenging problem^73^ Revisions after unsatisfactory adult circumcisions^60^ Rates of complications after newborn circumcision in a well‐baby nursery, special care nursery, and neonatal intensive care unit^29^
19. Meatitis	Examination of short and long term complications of thermocautery, plastic clamping, and surgical circumcision techniques^23^ Nontherapeutic circumcision of minors as an ethically problematic form of iatrogenic injury^4^ Complications of circumcision^27^ Male circumcision from an infectiological point of view^6^
20. Trapped/buried/concealed/inconspicuous penis	Examination of short and long term complications of thermocautery, plastic clamping, and surgical circumcision techniques^23^ Nontherapeutic circumcision of minors as an ethically problematic form of iatrogenic injury^4^ Dermatological complications of circumcision: Lesson learned from cases in a pediatric dermatology practice^61^ Complications of neonatal circumcision requiring surgical intervention in a developing country^40^ Inconspicuous penis^74^ Complications of circumcision^27^ Reconstruction of the penile skin loss due to ‘radical’ circumcision with a full thickness skin graft^31^ Buried and trapped penis: A case report^75^ Surgically correctable morbidity from male circumcision: Indications for specialist surgical care in Lagos^43^ The relationship between obesity and complications after neonatal circumcision^76^ Buried penis after newborn circumcision^77^ Rates of complications after newborn circumcision in a well‐baby nursery, special care nursery, and neonatal intensive care unit^29^ The inconspicuous penis in children^78^ Serious complications in male infant circumcisions in Scandinavia indicate that this always be performed as a hospital‐based procedure^32^
21. Urethrocutaneous fistula	Nontherapeutic circumcision of minors as an ethically problematic form of iatrogenic injury^4^ Complications of neonatal circumcision requiring surgical intervention in a developing country^40^ Complications of circumcision^27^ Multiple circumferential urethrocutaneous fistulae as a rare complication of circumcision and review of literature^79^ Penile injuries from proximal migration of the Plastibell circumcision ring^19^ Surgically correctable morbidity from male circumcision: Indications for specialist surgical care in Lagos^43^ Circumcision mishaps in Nigerian children^20^ Rates of complications after newborn circumcision in a well‐baby nursery, special care nursery, and neonatal intensive care unit^29^ Circumcision‐related tragedies seen in children at the Komfo Anokye Teaching Hospital, Kumasi, Ghana^38^ Penile epidermal inclusion cyst^80^
22. Iatrogenic hypospadias	Circumcision‐related tragedies seen in children at the Komfo Anokye Teaching Hospital, Kumasi, Ghana^38^ Nontherapeutic circumcision of minors as an ethically problematic form of iatrogenic injury^4^ Complications of circumcision^27^
23. Intraperitoneal bladder perforation (leading to life‐threatening renal failure)	Intraperitoneal bladder perforation and life‐threatening renal failure in a neonate following circumcision with the Plastibell device^81^
24. Circulatory shock	Serious complications in male infant circumcisions in Scandinavia indicate that this always be performed as a hospital‐based procedure^32^
25. Death (from bleeding or infection)	Newborn male circumcision^82^ Canadian Pediatrics Society position statement on newborn circumcision: A risk‐benefit analysis revisited^26^ A newborn with simmering bleeding after circumcision^47^ Serious complications in male infant circumcisions in Scandinavia indicate that this always be performed as a hospital‐based procedure^32^
26. Unsatisfactory cosmetic results including uncircumcised appearance	Newborn male circumcision^82^ Neonatal circumcision: New recommendations & implications for practice^1^ Revisions after unsatisfactory adult circumcisions^60^
27. Device displacement	Unexpected complications following adult medical male circumcision using the PrePex Device^83^ Penile injuries from proximal migration of the Plastibell circumcision ring^19^ Circumcision mishaps in Nigerian children^20^
28. Early sloughing of foreskin tissue	Unexpected complications following adult medical male circumcision using the PrePex Device^83^
29. Long foreskin obstructing urine flow	Unexpected complications following adult medical male circumcision using the PrePex Device^83^
30. Insufficient foreskin removal/redundant foreskin	Unexpected complications following adult medical male circumcision using the PrePex Device^83^ Acute ischemia of the glans penis after circumcision treated with hyperbaric therapy and pentoxifylline: Case report and revision of the literature^25^ Complications of circumcision^27^ Revisions after unsatisfactory adult circumcisions^60^ Buried and trapped penis: A case report^75^ Circumcision mishaps in Nigerian children^20^ Rates of complications after newborn circumcision in a well‐baby nursery, special care nursery, and neonatal intensive care unit^29^ Circumcision‐related tragedies seen in children at the Komfo Anokye Teaching Hospital, Kumasi, Ghana^38^ Serious complications in male infant circumcisions in Scandinavia indicate that this always be performed as a hospital‐based procedure^32^
31. (Acute) Ischaemia (which can lead to necrosis)	Acute ischemia of the glans penis after circumcision treated with hyperbaric therapy and pentoxifylline: Case report and revision of the literature^25^ Ischemia of the glans 24 hours after circumcision: A case report and therapeutic solution^84^ An unexpected complication: Glans ischemia after circumcision. Review of the literature^85^ Ischemia of the glans penis following circumcision: Case report and revision of the literature^36^
32. Pain	Acute ischemia of the glans penis after circumcision treated with hyperbaric therapy and pentoxifylline: Case report and revision of the literature^25^ Complications of circumcision^27^ Complications following circumcision: Presentations to the emergency department^28^ Serious complications in male infant circumcisions in Scandinavia indicate that this always be performed as a hospital‐based procedure^32^
33. Skin bridges (penile skin adhesion)	Neonatal circumcision: New recommendations & implications for practice^1^ Dermatological complications of circumcision: Lesson learned from cases in a pediatric dermatology practice^61^ Complications of circumcision^27^ Surgically correctable morbidity from male circumcision: Indications for specialist surgical care in Lagos^43^ The relationship between obesity and complications after neonatal circumcision^76^ Rates of complications after newborn circumcision in a well‐baby nursery, special care nursery, and neonatal intensive care unit^29^ Circumcision‐related tragedies seen in children at the Komfo Anokye Teaching Hospital, Kumasi, Ghana^38^
34. Fournier's gangrene	Fournier's gangrene‐delayed pedicle flap based upon the anterior abdominal wall^86^ Fournier's gangrene after adult male circumcision^87^
35. Penile mycosis leading to penile gangrene	Devastating penile mycosis leading to penile gangrene^88^
36. Glandular adhesion (of remnant foreskin)	Dermatological complications of circumcision: Lesson learned from cases in a pediatric dermatology practice^61^ Complications of neonatal circumcision requiring surgical intervention in a developing country^40^ Circumcision mishaps in Nigerian children^20^
37. Implantation dermoid/epidermal inclusion cysts/penile implantation cyst	Surgically correctable morbidity from male circumcision: Indications for specialist surgical care in Lagos^43^ Circumcision mishaps in Nigerian children^20^ Complications of neonatal circumcision requiring surgical intervention in a developing country^40^ Circumcision‐related tragedies seen in children at the Komfo Anokye Teaching Hospital, Kumasi, Ghana^38^ Nontherapeutic circumcision of minors as an ethically problematic form of iatrogenic injury^4^ Dermatological complications of circumcision: Lesson learned from cases in a pediatric dermatology practice^61^ Penile epidermal inclusion cyst^80^ Complications of circumcision^27^ Penile epidermal inclusion cyst^89^ Epidermal inclusion cyst as a rare complication of neonatal male circumcision: A case report^90^
38. Myiasis (fly infestation)	Myiasis as a rare complication of male circumcision: A case report and review of literature^91^
39. Denuded penis	Penile resurfacing for denuded penis following circumcision^37^
40. Chordee	Complications of circumcision^27^ Circumcision mishaps in Nigerian children^20^
41. Suture sinus tracts	Complications of circumcision^27^
42. Lower clinical neurophysiological elicitability of the penilo‐cavernosus reflex	Clinical elicitation of the penilo‐cavernosus reflex in circumcised men^92^
43. Sexual dysfunction	Reconstruction of the penile skin loss due to ‘radical’ circumcision with a full thickness skin graft^31^
44. Traumatic neuroma	Traumatic neuroma of the penis after circumcision—Case report^93^
45. Decreased urine output	Complications following circumcision: Presentations to the emergency department^28^
46. Excessive skin removal	Rates of complications after newborn circumcision in a well‐baby nursery, special care nursery, and neonatal intensive care unit^29^ Serious complications in male infant circumcisions in Scandinavia indicate that this always be performed as a hospital‐based procedure^32^
47. Injury to urethra	Rates of complications after newborn circumcision in a well‐baby nursery, special care nursery, and neonatal intensive care unit^29^ Serious complications in male infant circumcisions in Scandinavia indicate that this always be performed as a hospital‐based procedure^32^

## 4 DISCUSSION

Male circumcision is a common procedure performed in many parts of the world by many different types of providers. A wide variety of complications arising from circumcision surgeries are reported in the literature. Forty‐seven different complications that arose from male circumcision surgery were found in this study. This does not imply that every male circumcision performed results in a complication but it is important to note that there can be serious life‐altering consequences from this procedure, even if it is done correctly. Some of these complications are relatively minor and simple to treat; others, however, require additional surgery to correct the complication. But some complications are irreversible. Even psychological issues have been reported to arise in children after operations, including circumcisions.^17^ These mental post‐operative issues are not commonly listed as potential complications on informed consent literature and are not often taken into account as a risk of circumcision.

More complications arise if the circumcision operation is done after the neonatal period, if it does not respect strict antisepsis, if it is performed by a less experienced provider and if no clinical investigation is performed for identification of genetic bleeding defects.

While experienced providers who practice in sterile settings have better outcomes with fewer complications, encouraging parents to take into account who is performing their son's circumcision, what was their training, how clean is their practice and how much experience they have and reminding them they have the option to decline the procedure entirely allow the parents to get a more complete picture and play an essential role in the decision‐making process.

Healthcare providers, especially surgeons who perform male neonatal circumcisions, need to be informed of the comprehensive list of potential complications of the surgery. Understanding the extent to which such complications occur will allow them to provide more detailed, factual and balanced information to parents regarding the potential risks, including short‐ and long‐term complications, many of which are either reversible through additional surgery or are permanent. As such, providers should plan in advance for potential complications and explain to parents what their next steps should be if such a complication were to arise. Parents should also be sure to ask their healthcare provider about a number of issues: If complications do arise, what plans are in place to minimize them as quickly as possible? Do providers have immediate access to clotting factors in case the baby will not stop bleeding? Do they have an arrangement with neonatologists in their hospital to consult in such cases? Do they have multiple instruments at the ready that they are trained and comfortable in using? Will they pick the technique of circumcision that best fits the baby's anatomy?

Given the results of this study, it is clear that physicians must be prepared to counsel parents who are unsure of the decision to circumcise their sons or not, taking into account the vast number of complications that could arise as a result. The medical professionals must put aside their own personal beliefs and experiences in order to listen to the patient and parents' experiences, concerns and hesitations. Some families may come in with a decision already made about wanting to get the procedure done, and because parents may have a number of religious or cultural reasons for wanting their infant circumcised, it is vital that providers be able to participate in a balanced discussion that also takes into account the various types of complications and their potential severity. But it is also likely that a family will come into the office unsure of what to do. The physician must step up and counsel them. It is not ethical to push onto a patient or his family a procedure that they are unsure or uninformed about. Other families will have already decided to not circumcise their sons. Their decisions must be respected.

It is equally important that physicians discuss with their patients and parents ways of addressing common issues that could develop in natural male anatomy without the use of surgical intervention, for example, genital hygiene and presence of phimosis in a prepubescent male. Phimosis is present in almost all neonates, and the prepuce is eventually retractable in 99% of males by age 16.^15^, ^16^ If a prepubescent patient presents with tight but otherwise normal foreskin, watching and waiting until after puberty should be considered.^15^

Male circumcision is not without its risks and complications, and all families, regardless of hesitations or not, must be informed of these potential adverse effects. In any scenario, a well‐researched and experienced physician who can offer guidance and counselling will provide patients and their families comfort and guidance through this irreversible decision.

## CONFLICT OF INTEREST

No interests to declare.

## AUTHOR CONTRIBUTIONS

Stanca Iris Iacob did the background research and wrote the text. Lauren Sardi gave guidance as to what topic could be covered in a systematic review, guided the initial phase of background research and helped with editing the text. Richard S. Feinn had the idea of creating the tables, helped format them and incorporated them into the text.

## Supporting information


**Figure S1.** Supporting Information
