# Supplementary material for: Systematic review of complications arising from male circumcision
Source: BJUI Compass. 2021 Nov 11;3(2):99–123. doi: 10.1002/bco2.123 (PMC8988744; doi:10.1002/bco2.123)
Supplement: Supplementary file 1 — Figure S1. Supporting Information [file BCO2-3-99-s001.doc]

**
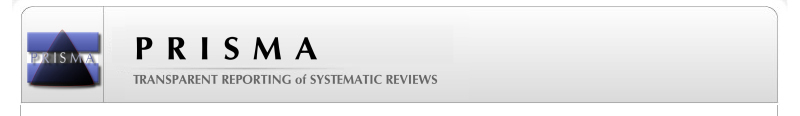
PRISMA 2009 Flow Diagram**

**Screening**

**Included**

**Eligibility**

**Identification**

Articles published from 1945-2020
identified by searching “male circumcision” on PubMed, on June 16^th^, 2020

(n = 6,641)

Articles published from 2000-2020
identified by searching “male circumcision” on PubMed, on June 16^th^, 2020

(n = 4,464)

Full-text articles excluded for not mentioning complications from male circumcision surgeries
(n = 4,330)

Full-text articles referring to specific complications from male circumcision surgeries
(n = 134)

Full-text articles excluded for not mentioning specific complications from male circumcision surgeries

(n = 56)

Articles used to compile a list of 47 specific complications arising from male circumcisions

(n = 78)
